# ID 274 - Eficácia e Segurança das Estratégias Terapêuticas para a Brucelose Humana: uma revisão sistemática e metanálise de rede

**DOI:** 10.5327/2237-9622.2025.v34s1.274

**Published:** 2025-11-25

**Authors:** Endi Lanza Galvão, Gláucia Cota, Diego Mendes Xavier, Glaciele Maria de Souza, Marina Rocha Fonseca Souza, Moisés Willian Aparecido Gonçalves, Felipe Francisco Bondan Tuon, Sarah Nascimento Silva

**Keywords:** brucelose humana, eficácia, revisão sistemática, metanálise em rede

## Abstract

**Introdução:**

A brucelose humana é uma doença negligenciada, reemergente e endêmica em muitos países. Trata-se de uma zoonose com transmissão associada à manipulação de animais e à ingestão de produtos contaminados. A doença geralmente se apresenta como um quadro febril subagudo a crônico caracterizado por sintomas inespecíficos, tais como astenia, perda de peso e sudorese. Esquemas terapêuticos combinando dois ou mais medicamentos têm se mostrado superiores à monoterapia. O potencial debilitante e incapacitante alerta quanto à morbidade da doença, cujos sintomas podem persistir por semanas ou meses, gerando impacto socioeconômico. A evidência até agora acumulada é baseada em ensaios clínicos que compararam as intervenções de interesse contra diferentes comparadores. O objetivo deste estudo foi atualizar o estado atual da evidência sobre a eficácia e a segurança das opções terapêuticas para a brucelose humana utilizando a abordagem metanálise em rede, para embasar o Protocolo Clínico e Diretrizes Terapêuticas (PCDT) da Brucelose Humana no Brasil.

**Método:**

Uma busca sistemática foi conduzida nas bases MEDLINE (PubMed), Embase, (CENTRAL) e Biblioteca Virtual de Saúde (BVS) por revisores independentes para avaliar insucesso da terapia, eventos adversos e tempo até a defervescência associado às diferentes terapias. Foram selecionados ensaios clínicos randomizados (ECRs) avaliando qualquer intervenção terapêutica medicamentosa, sendo excluídos estudos não originais, ou relacionados às formas localizadas da doença, ou com menos de dez participantes. O risco de viés foi avaliado pela Ferramenta Revisada para Avaliação do Risco de Viés em Ensaios Clínicos Randomizados (RoB 2.0). Os dados foram analisados por metanálise em rede frequentista usando modelo de efeitos randômicos. A certeza da evidência foi avaliada usando a abordagem (CINeMA). O protocolo foi registrado no PROSPERO (CRD42023411952).

**Resultados:**

Foram incluídos 31 ECRs envolvendo 4.167 pacientes. Três redes de evidências foram identificadas para avaliação dos desfechos de interesse. A terapia tripla com doxiciclina + estreptomicina + hidroxicloroquina por 42 dias (RR 0,08; IC 95% 0,01-0,76) apresentou menor risco de insucesso comparada ao esquema doxiciclina + estreptomicina. O esquema doxiciclina + rifampicina apresentou maior risco de insucesso quando comparado à terapia com doxiciclina + estreptomicina (RR 1,96; IC 95% 1,27-3,01). Nenhuma diferença significativa foi observada entre os esquemas na análise da incidência de eventos adversos e tempo até a defervescência. Em geral, a maioria dos estudos apresentou alto risco de viés e os resultados foram muito baixa certeza da evidência.

**Conclusão:**

Nesta revisão, a superioridade de medicamentos já indicados para o tratamento da brucelose humana, como a combinação de doxiciclina e aminoglicosídeos, foi confirmada. A inclusão de hidroxicloroquina ao esquema duplo mostrou-se uma estratégia promissora para reduzir o risco de insucesso terapêutico, embora seja necessária confirmação em estudos futuros. Os resultados desta revisão subsidiaram a elaboração do PCDT para a Brucelose Humana no Brasil, estabelecendo informações essenciais para as recomendações do tratamento clínico. Especificamente, parte dos dados analisados foi utilizada para apoiar a incorporação do sulfato de gentamicina combinado à doxiciclina, ampliando o arsenal terapêutico para o tratamento da brucelose humana no SUS.

